# Supplementary material for: MetaRibo-Seq measures translation in microbiomes
Source: Nat Commun. 2020 Jun 29;11:3268. doi: 10.1038/s41467-020-17081-z (PMC7324362; doi:10.1038/s41467-020-17081-z)
Supplement: Supplementary file 10 — Supplementary Data 7 [file 41467_2020_17081_MOESM10_ESM.zip › File2/Confidence_High_Taxonomy/346353_out.krona.html]

Javascript must be enabled to view this page.

members
magnitude
magnitudeUnassigned
count
unassigned
taxon
rank

346353\_out

14

superkingdom
14
2

phylum
976
14

class
14
200643

order
14
171549

family
815
2

2
816
genus

820

SRS148319\_contig\_number\_134
1
species

246787
1

SRS077086\_contig\_number\_7684
species

12
2005525
family

12
375288
genus

species
46503
12

SRS013476\_contig\_number\_contig-100\_1362.222193SRS016132\_contig\_number\_contig-100\_1169.44435SRS018936\_contig\_number\_3204SRS019161\_contig\_number\_12504SRS062654\_contig\_number\_contig-100\_919.151337SRS065397\_contig\_number\_9909SRS077392\_contig\_number\_42519SRS1041116\_contig\_number\_5986SRS143780\_contig\_number\_27198SRS146764\_contig\_number\_32865SRS148721\_contig\_number\_20016SRS893378\_contig\_number\_9806
